# Gene status and clinicopathologic characteristics of lung adenocarcinomas with mediastinal lymph node metastasis

**DOI:** 10.18632/oncotarget.11494

**Published:** 2016-08-22

**Authors:** Shumeng Zhang, Bing Yan, Jing Zheng, Jing Zhao, Jianying Zhou

**Affiliations:** ^1^ Department of Respiratory Disease, Thoracic Disease Center, The First Affiliated Hospital, College of Medicine, Zhejiang University, Hangzhou 310003, China; ^2^ Department of Pathology, The First Affiliated Hospital, College of Medicine, Zhejiang University, Hangzhou 310003, China

**Keywords:** adenocarcinoma of the lung, EGFR, ALK, ROS1, RET

## Abstract

Lung cancer with mediastinal lymph node metastasis is more likely to develop recurrence and metastasis after complete resection and targeted therapy is a promising treatment strategy. We performed amplification refractory mutation system (ARMS) fluorescence quantitative PCR to detect the gene status of EGFR, ALK, ROS1 and RET in resected samples from 280 patients who were confirmed to have primary lung adenocarcinomas with N1-N2 lymph node metastasis. Of the 280 patients enrolled, the frequency of EGFR mutations, ALK fusions, ROS1 fusions, RET fusions and no mutations was 42.9%, 10.7%, 1.8%, 3.6% and 42.9%, respectively. Five patients exhibited the coexistence of the EGFR and ALK alterations. ALK, ROS1 and RET fusions were mutually exclusive. The frequency of EGFR mutation was significantly lower among patients with poor differentiation, while the rates of ALK and ROS1 fusions were the opposite. RET fusions also tended to be more prevalent in poorly differentiated patients. EGFR and ALK double positive tumors were characterized by significantly smaller size compared with those had single gene alteration. Our study comprehensively analyzed the distinct and common clinicopathologic characteristics according to genotypes of the cohort, which should help in categorizing patients for efficient screening.

## INTRODUCTION

Although diagnosis at an early stage is increasing and therapeutic methodology is progressing, lung cancer remains the leading cause of cancer-related deaths worldwide [1]. Over the last decade, great achievements have been made in our understanding of lung cancer. With the identification of oncogenic driver mutations, the therapy for non-small cell lung cancer (NSCLC), especially lung adenocarcinoma has undergone a revolutionary change [2].

Epidermal growth factor receptor tyrosine kinase inhibitors (EGFR-TKIs), such as gefitinib and erlotinib, have been used against advanced NSCLCs with EGFR mutations, demonstrating remarkable improvements in patient outcome. Crizotinib, the ALK/MET TKI, has shown dramatic therapeutic effects against lung cancer with anaplastic lymphoma kinase (ALK) rearrangements. More recently, ROS1 and RET fusions have attracted much attention, as they were both identified in approximately 1%-2% of patients with NSCLC. Crizotinib has been approved by FDA for the treatment of metastatic ROS1 positive NSCLC. Clinical trials are underway to investigate the therapeutic effects of cabozantinib and vandetanib against NSCLC harboring RET fusions [3]. All of these achievements highlight the importance of matching targeted therapy to the genetically defined subgroups of patients. However, the frequency of these alterations, especially the fusion genes is comparatively low. Thus, identifying the enriched population is of significance for efficient screening. Previous studies have reported the clinical and pathologic features according to genotype. However, the opinions remain controversial. This may be due to the different nature of the study cohorts, as many of the cohorts are unselected NSCLCs or adenocarcinomas. Moreover, comprehensive studies of alteration-specific and common clinicopathologic features are rare [4].

As far as we know, the extent of lymph node involvement in NSCLCs is the most important prognostic factor and influences treatment strategy [5]. NSCLC with lymph node metastasis is more likely to develop recurrence and metastasis after surgical resection and have a shorter survival time after recurrence [6]. It is necessary to test the gene status of NSCLCs after resection with regional lymph node metastasis. Targeted therapy has changed the therapeutic strategy for advanced-stage NSCLC, and interest in moving targeted therapy from advanced-stage patients to early-stage patients has triggered a heated debate. Studies are currently underway, including the SELECT, RADIANT and ALCHEMIST studies [7, 8]. However, the status of targeted therapy in adjuvant treatment is controversial. Even so, recurrence and metastasis do occur, and many patients are potential candidates for targeted therapy.

To gain a comprehensive understanding of oncogenic driver mutations and their associated clinicopathologic characteristics of this special cohort, we concurrently investigated EGFR, ALK, ROS1 and RET alterations in samples from 280 lung adenocarcinoma patients with N1-N2 lymph node metastasis and analyzed the common and specific clinical features of different molecular alterations.

## RESULTS

### Patient demographics and clinicopathologic characteristics

The cohort comprised 280 patients, among which 133 (47.5%) were male and 147 (52.5%) were female, ranging in age from 31 to 87 years (median, 60 years). Approximately 94 (33.6%) were smokers and 186 (66.4%) were nonsmokers. Out of the lung cancers, 43 (15.4%) were staged by the 7th TNM staging system as stage IIA, 62 (22.1%) as stage IIB, 156 (55.7%) as stage IIIA, 5 (1.8%) as IIIB, and 14 (5.0%) as stage IV. Approximately 52 (18.6%) tumors were histologically classified as having poor differentiation, 170 (60.7%) as having poor-moderate differentiation, 51 (18.2%) as having moderate differentiation, and 3 (1.1%) as having moderate-good differentiation; the others were undetermined. A total of 152 (54.3%) tumors were ≤ 3 cm and 128 (45.7%) were > 3 cm; 123 (43.9%) patients had N1 lymph node involvement and 157 (56.1%) had N2 lymph node involvement. The demographic data and characteristics of the study population were listed in Table 1.

**Table 1:** Baseline characteristics of patients and tumors

Characteristics	N	%
**Age (years)**		
Mean ±SD	59.5 ±9.6	
Median (Range)	60 (31-87)	
**Gender**		
Male	133	47.5
Female	147	52.5
**Smoking status**		
Smoker	94	33.6
Non-smoker	186	66.4
**Tumor size (cm)**		
Median (Range)	3 (1-12)	
≤ 3 cm	152	54.3
> 3 cm	128	45.7
**Stage**		
IIA	43	15.4
IIB	62	22.1
IIIA	156	55.7
IIIB	5	1.8
IV	14	5.0
**Differentiation**		
Poor	52	18.6
Poor-moderate	170	60.7
Moderate	51	18.2
Moderate-good	3	1.1
Undetermined	4	1.4
**N stage**		
N1	123	43.9
N2	157	56.1

### The mutation status and coexistence of EGFR, ALK, ROS1 or RET

Among the patients with lung adenocarcinomas with lymph node metastasis, the overall frequencies were as follows: EGFR mutations, 42.9% (120/280); ALK fusions, 10.7% (30/280); RET fusions, 3.6% (10/280); ROS1 fusions, 1.8% (5/280); and no mutations (wild type[WT]) in any of the 4 genes (WT/WT/WT/WT), 42.9% (120/280). In the 120 patients with EGFR mutations, 49.2% (59/120) of mutations were exon 19 deletions and 46.7% (56/120) of mutations were exon 21 mutations, accounting for 95.8% of all EGFR mutations. Among the patients with EGFR mutations or ALK, RET, ROS1 fusions, five (5/280, 1.8%) patients exhibited the coexistence of the EGFR mutations (3 with exon 19 deletions, 2 with exon 21 mutations) and the ALK fusions. No coexistence of ALK, ROS1 or RET was observed, as shown in Table 2.

**Table 2:** The genotypes of 280 lung adenocarcinomas with lymph node metastasis

Genotype	N	%
EGFR mutations	120	42.9
21	56	
20	2	
19	59	
18	1	
19+T790M	1	
19+21	1	
ALK fusions	30	10.7
ROS1 fusions	5	1.8
RET fusions	10	3.6
WT/WT/WT/WT	120	42.9
coexistence of EGFR and ALK	5	1.8

### Association between clinicopathologic features and EGFR mutations

The clinicopathologic characteristics of the cases with EGFR mutations, ALK, ROS1 or RET fusions were listed in Table 3 and Supplementary Table S1.

**Table 3:** Clinicopathologic characteristics of lung adenocarcinomas with lymph node metastasis harboring EGFR mutations or ALK, ROS1, RET fusions

Characteristics	EGFR(N=120)			ALK(N=30)			ROS1(N=5)			RET(N=10)		
	Positive (n=120)	Negative (n=160)	*P*	Positive (n=30)	Negative (n=250)	*P*	Positive (n=5)	Negative (n=275)	*P*	Positive (n=10)	Negative (n=270)	*P*
**Age (years)**			0.388			**0.038**			1.000			0.752
≤ 60	59	87		21	125		3	143		6	140	
> 60	61	73		9	125		2	132		4	130	
**Gender**			**0.030**			0.923			1.000			0.341
**Male**	48	85		14	119		2	131		3	130	
**Female**	72	75		16	131		3	144		7	140	
**Smoking history**			0.273			0.397			1.000			0.504
Smoker	36	58		8	86		2	92		2	92	
Non-smoker	84	102		22	164		3	183		8	178	
**Tumor size (cm)**			0.835			0.912			1.000			0.353
≤ 3	66	86		16	136		3	150		7	145	
> 3	54	74		14	114		2	125		3	125	
**TNM stage**			0.170			0.704			1.000			1.000
IIA-IIIA	109	152		29	232		5	256		10	251	
IIIB-IV	11	8		1	18		0	19		0	19	
**Differentiation**			**0.003^a^**			**0.024^a^**			**0.003^a^**			0.257^a^
Poor	10	42	Ref.	10	42	Ref.	4	48	Ref.	3	49	
Poor-moderate	81	89	<0.001	17	153	0.075	1	169	0.011	5	165	
Moderate	24	27	**0.003**	3	48	**0.041**	0	51		1	50	
Moderate-good	2	1	0.117	0	3		0	3		0	3	
Undetermined	3	1		0	4		0	4		1	3	
**N stage**			0.862			0.749			1.000			0.194
N1	52	71		14	109		2	121		2	121	
N2	68	89		16	141		3	154		8	149	

Bold represents statistically significance, *P* <0.05

a *P* values refer to overall comparisons across all subgroups except the undetermined

**Figure 1:**
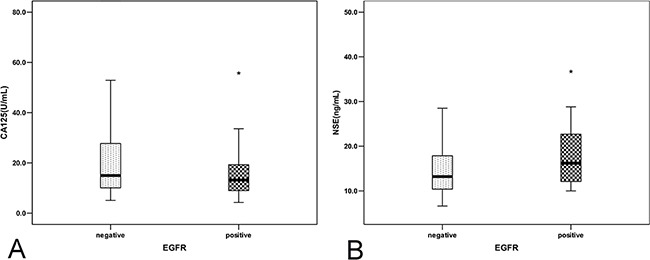
EGFR mutation and tumor marker **A.** The carbohydrate antigen 125 (CA125) level of the EGFR mutation group and the wild-type group; *P<0.05 vs wild-type group. **B.** The neuro specific enolase (NSE) level of the EGFR mutation group and the wild-type group; *P<0.05 vs wild-type group.

**Figure 2:**
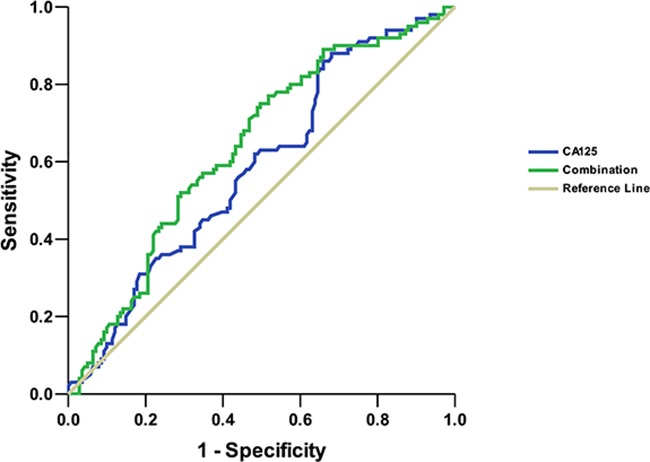
Receiver operating characteristics (ROC) curve analysis for CA125 level and a combination of CA125, gender and differentiation as predictors of EGFR mutations in lung adenocarcinomas with lymph node metastasis

The frequency of EGFR mutations was higher in females than in males (49.0% vs. 36.1%, *P*=0.030). The EGFR mutation rate was lower among patients with poor differentiation (19.2%, [10/52]) compared with those with poor-moderate differentiation (47.6%, [81/170]) (*P*<0.001), moderate differentiation (47.1%, [24/51]) (*P*=0.003), and moderate-good differentiation (66.7%, [2/3]) (*P*=0.117) (Table 3). The carbohydrate antigen 125 (CA125) level of the EGFR mutation group was lower than that of the wild-type group (*P*=0.025), while the neuro specific enolase (NSE) level of the EGFR mutation group was higher than that of the wild-type group (*P*=0.038) (Figure 1). In multivariate logistic regression, differentiation (poor/moderate differentiation, OR 0.035, 95%CI 0.002-0.508, *P*=0.014) and CA125 level (OR 0.888, 95%CI 0.799-0.988, *P*=0.029) were independently associated with EGFR mutation, while gender (female/male, OR 1.404, 95%CI 0.309-6.377, *P*=0.660) and NSE (OR 1.094, 95%CI 0.963-1.241, *P*=0.166) were not. We also plotted the Receiver operating characteristics (ROC) curve for serum CA125 as a predictor of EGFR. The area under the ROC curve (AUC) was 0.585 (95%CI, 0.513-0.657) and the optimal cutoff point for CA125 in the cohort was 21.85U/ml (sensitivity of 86.0% and specificity of 34.0%). In combination with gender and differentiation, the AUC was elevated to 0.638 (95%CI, 0.568-0.708) (Figure 2).

### Association between clinicopathologic features and ALK fusions

ALK fusions occurred more frequently in young patients (≤60 years old) (*P*=0.038). The frequency of ALK fusions was higher among patients with poor differentiation (19.2%, [10/52]) compared to those with poor-moderate differentiation (10.0%, [17/170]) (*P*=0.075) and moderate differentiation (5.9%, [3/51]) (*P*=0.041). The incidence of ALK fusions was higher in EGFR wild-type patients than that of the EGFR mutation patients (15.6% vs. 4.2%, *P*=0.002). (Table 3 and Supplementary Table S1). Subsequent multivariate logistic regression demonstrated that EGFR status (mutation/wild-type, OR 0.280, 95%CI 0.101-0.774, *P*=0.014) were independent predictive factors for ALK fusions.

### Association between clinicopathologic features and ROS1 fusions

ROS1 fusions were more prevalent in patients with poor differentiation (*P*=0.011). Patients with ROS1 fusions had significantly lower levels of CA199 and NSE compared with the wild-type group (*P*=0.030, *P*=0.004)(Table 3 and Supplementary Table S1).

### Association between clinicopathologic features and RET fusions

RET fusions were observed more frequently in patients with poor differentiation (5.8%, [3/52]) compared with those with poor-moderate differentiation (2.9%, [5/170]) and moderate differentiation (2.0%, [1/51]); however, these differences had no statistical significance (all *P*>0.05) (Table 3 and Supplementary Table S1).

### Comparison of the clinicopathologic features between EGFR mutations and ALK/ROS1/RET fusions

**Figure 3:**
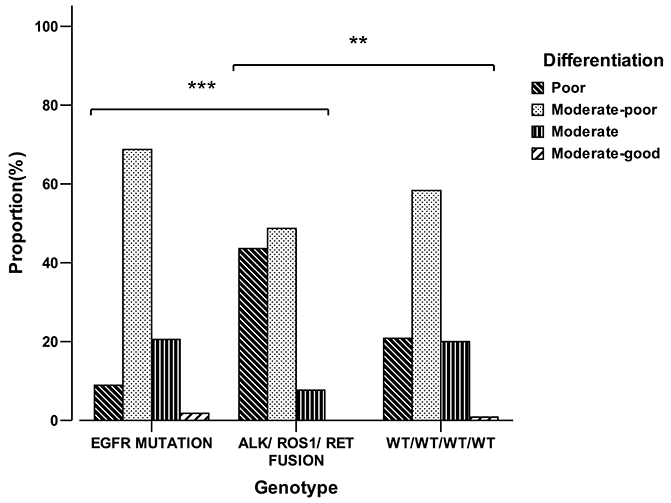
Comparison of differentiation between EGFR mutation, ALK/ROS1/RET fusion and wild type group **P<0.01, ***P<0.001 vs ALK/ROS1/RET fusion group.

On the basis of the genotype, the patients were classified into three distinct groups: the EGFR group, the fusion (ALK/ROS1/RET) group and the WT/WT/WT/WT group. The five patients who exhibited the coexistence of ALK fusions and EGFR mutations were not included. The differentiation of the fusion group was significantly different from that of the EGFR group and WT/WT/WT/WT group (*P*<0.001, *P*=0.003) (Figure 3). In patients with poor differentiation, the rate of fusions (ALK/ROS1/RET) was 32.7% (17/52), tended to be higher than that of EGFR mutation 19.2% (10/52), however, with no statistical significance (*P*=0.117). Females were more popular in EGFR group compared with the WT/WT/WT/WT group (*P*=0.011) (Supplementary Table S2).

### Clinical features of patients harboring EGFR mutations and ALK fusions

**Figure 4:**
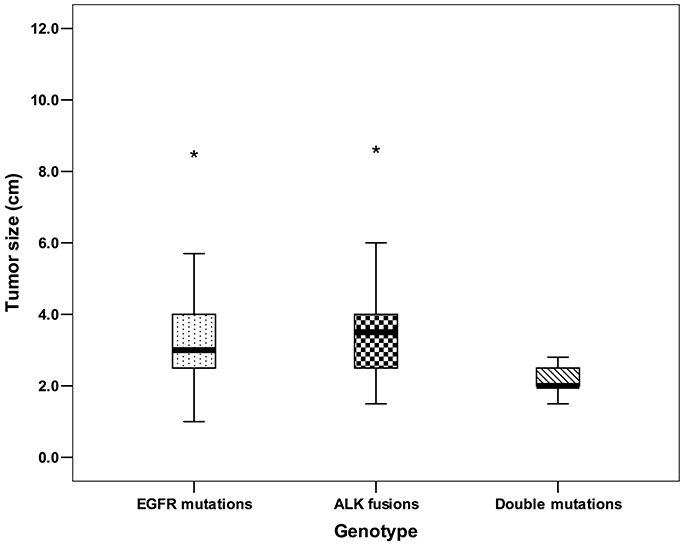
Comparison of tumor size between patients with double mutations and single gene mutations *P<0.05 vs patients with double mutations.

We compared the clinicopathologic features of the five patients harboring both EGFR mutations and ALK fusions with that of patients with single-gene mutations. The tumor sizes of the five patients were all ≤ 3 cm and significantly smaller than those of patients with either EGFR mutations or ALK fusions(*P*=0.017, *P*=0.011)(Figure 4, Supplementary Table S3).

## DISCUSSION

Here, we performed a comprehensive analysis of the correlation between the well-identified driver mutations and clinical characteristics of the lung adenocarcinomas with mediastinal lymph node involvement. We identified that the frequencies of EGFR, ALK, RET and ROS1 were 42.9%, 10.7%, 3.6% and 1.8%, respectively. The rate of ALK fusions was higher than reported. In addition, we found that the tumors exhibited mainly poor and poor-moderate differentiation, which was consistent with previous studies [9, 10].

In the present study, we found that the incidence of EGFR mutations was associated with female sex in univariate analysis instead of multivariate analysis, which was in concordance with previous studies [11]. Previous studies reported that ALK-positive patients were more likely to be younger and with higher grade tumors. It appeared to be associated with a higher risk of progression, recurrence and metastases [12, 13]. One study reported that gender, smoking history and N stage were independently associated with ALK fusions [14]. In our study, N0 and N3 patients were not enrolled, and there was no significant difference of the ALK fusions rate between N1 and N2 patients. We also found that ALK-positive patients were younger, which was consistent with previous studies. Patients with ROS1 fusions shared some features in common with ALK-positive patients in previous studies, such as younger age, female sex, never-smoking status, adenocarcinoma, advanced stage and Asian ethnicity [15, 16]. Previous studies revealed that RET fusions were more common among never-smokers and those with adenocarcinoma histology, and the tumors tended to be small in size, poorly differentiated, and represent N2 disease [17]. Despite similar clinicopathologic features, there was no evidence of the coexpression of ALK, ROS1 and RET in the present study.

Our study showed that the EGFR mutation rate was significantly lower among patients with poor differentiation, while the rates of ALK and ROS1 fusions were the opposite. RET fusions also tended to be more prevalent in poorly differentiated patients. In the poorly differentiated patients, the frequency of ALK/ROS1/RET fusions was comparable with EGFR mutations. These findings suggested that in poorly differentiated patients, the fusion genes should be put on the comparable status with EGFR mutations when selecting enriched populations for screening. KRAS, another well-identified oncogene, was reported to have a mutation rate of 8.3% in Chinese population. In addition, KRAS positive patients were more likely to have poorer differentiation [18], which suggests that test of KRAS mutation should be performed, especially in poorly differentiated NSCLC patients. Although there haven't been any therapeutic approaches for KRAS-positive lung cancer approved, mutant KRAS is still a promising potential therapeutic target in the high-risk population.

Previous studies reported that serum CEA levels were associated with EGFR mutations in patients with lung adenocarcinomas [19]. However, our study did not show a correlation of CEA levels with neither EGFR mutations, nor with ALK, ROS1 or RET fusions. Moreover, we found that a lower CA125 level was independently associated with a higher rate of EGFR mutations. The predictive value of a CA125 level on EGFR mutations was limited when it was applied alone, but could be improved when it was combined with other clinical characteristics. One possible explanation is that CA125 is a non-specific tumor marker which can be easily influenced by many different factors. A large series were still required to evaluate the value of a CA125 level on EGFR mutations.

EGFR and ALK are generally considered to be largely mutually exclusive [20, 21]. However, several recent studies identified that EGFR mutations and ALK fusions coexist [22–24]. Our findings showed that EGFR mutations were independent predictive factors for ALK fusions. However, the coexistence of EGFR mutations and ALK fusions were still detected in five cases. EGFR and ALK double positive tumors were characterized by significantly smaller size compared with those had either EGFR or ALK alterations. The finding has not been reported in previous studies.

Our study comprehensively and concurrently analyzed the four oncogenic alterations of adenocarcinomas with mediastinal lymph nodes involvement, which was different from previous studies. However, after the mutation profiling and separation into each subgroup, the number of the samples in each subgroup became quite small. Some possibly existed minor features might be overlooked. In our study, the mutation status was examined by ARMS-PCR. PCR-based method was simple, high-throughput and with high accuracy. But it can't detect unknown mutations, which would result in a risk of missing some important mutations and their associations. In this retrospective study, the survival analysis was not carried out due to immature survival data, which was its main limitation.

In summary, in the cohort of lung adenocarcinoma with mediastinal lymph node metastasis, differentiation was mainly poor. The coexistence of ALK and EGFR was shown, while ALK, ROS1 and RET was demonstrated to be mutually exclusive. Our study comprehensively analyzed the distinct and common clinicopathologic characteristics according to genotypes of the cohort, which should help in categorizing patients for efficient screening.

## MATERIALS AND METHODS

### Patients and samples

From Jan 2011 to Aug 2013, we consecutively enrolled patients who underwent complete surgical resection and were diagnosed as lung adenocarcinomas with mediastinal lymph node metastasis at the First Affiliated Hospital of College of Medicine, Zhejiang University. No patient had received preoperative therapy, such as chemotherapy, radiotherapy or biotherapy. Formalin-fixed and paraffin-embedded (FFPE) samples of completely resected lung adenocarcinomas were used. The histologic differentiation were evaluated and confirmed by expert lung pathologists according to the WHO classification criteria for lung cancer: lepidic growth was graded as good differentiation, acinar and papillary patterns as moderate differentiation, solid and micropapillary morphology as poor differentiation. Moderate-good differentiation was between moderate and good differentiation. Poor-moderate differentiation was between poor and moderate differentiation. The staging was according to the 7th edition of the TNM classification for lung cancer.

Medical records of all patients were reviewed, including age, gender, smoking history, tumor size, TNM stage and preoperative tumor markers, such as the serum carcinoembryonic antigen (CEA) level. Never-smokers were defined as patients who had a smoking exposure of < 100 cigarettes in their lifetime. The clinical and pathological characteristics were reviewed and assessed according to driver oncogenes. The study was approved by the Ethics Committee of the First Affiliated Hospital of College of Medicine, Zhejiang University. The Ethics Committee waived the need for consent for use of the samples in research.

### Detection of EGFR mutations and ALK, ROS1 and RET fusions

With the routine diagnosis of lung adenocarcinoma with lymph node metastasis established, the residual material of the FFPEs was screened for molecular analysis. Genomic DNA and total RNA were extracted with the Qiagen QIAamp DNA FFPE Tissue Kit and Qiagen RNeasy FFPE Kit (Qiagen, Germany). mRNA was transcribed to cDNA. The status of the EGFR, ALK, ROS1 and RET genes was detected by ARMS-PCR using commercially available kits from Amoy Diagnostics (Xiamen, China), namely, the ADx EGFR Mutation Diagnostic Kit, ADx EML4-ALK Fusion Gene Diagnostic Kit, ADx ROS1 Fusion Gene Diagnostic Kit and ADx RET Fusion Gene Diagnostic Kit on a Stratagene Mx3000P QPCR System (Agilent Technologies, Santa Clara, CA, USA) [25].

### Statistical analysis

The correlations between gene mutations and clinicopathologic variables were analyzed. The normally distributed variables were characterized by mean and standard deviation, while nonparametric distributed variables were characterized by median and interquartile ranges. Pearson's test and Fisher's exact test were used to compare categorical variables, along with Student's t test and ANOVA for numerical variables. The Mann-Whitney U and Kruskal-Wallis H tests were performed to assess the differences between nonparametric distributed variables and ordered categorical variables. Factors with *P* less than 0.05 in the univariate analysis were further analyzed by multivariate logistic regression. All tests were two-tailed, and a *P* value of <0.05 was set as statistically significant. Statistical analyses were performed with SPSS version 19.0.

## SUPPLEMENTARY TABLES




