# Supplementary material for: Gene status and clinicopathologic characteristics of lung adenocarcinomas with mediastinal lymph node metastasis
Source: Oncotarget. 2016 Aug 22;7(39):63758–66. doi: 10.18632/oncotarget.11494 (PMC5325401; doi:10.18632/oncotarget.11494)
Supplement: Supplementary file 1 [file oncotarget-07-63758-s001.pdf]

Gene status and clinicopathologic characteristics of lung adenocarcinomas with mediastinal lymph node metastasis

SUPPLEMENTARY TABLES

Supplementary Table S1: Tumor marker and gene status.

See Supplementary File 1

Supplementary Table S2: Comparison of clinicopathologic features between EGFR mutations and ALK/ROS1/RET fusions

Characteristics	EGFR N=115	FUSION (ALK/ ROS1/RET) N=40	WT/WT/WT/WT N=120	*P*
**Age (years)**				0.103
≤ 60	56	27	60	
> 60	59	13	60	
**Gender**				**0.030**
Female	69	23	52	
Male	46	17	68	
**Smoking history**				0.112
Smoking	34	10	48	
Never smoking	81	30	72	
**Tumor size (cm)**				0.940
≤ 3	61	21	66	
> 3	54	19	54	
**Tumor stage**				0.316
IIA-IIIA	104	39	113	
IIIB-IV	11	1	7	
**Differentiation**				**<0.001**[a]
Poor	10	17	25	
Poor-moderate	77	19	70	
Moderate	23	3	24	
Moderate-good	2	0	1	
Undetermined	3	1	0	
**N stage**				0.574
N1	49	15	56	
N2	66	25	64	

Bold represents statistically significance, $P < 0.05$

[a] *P* values refer to overall comparisons across all subgroups except the undetermined

Supplementary Table S3: Clinical features of patients harboring EGFR mutations and ALK fusions

Characteristics	EGFR mutations	ALK fusions	Double mutations	*P* [a]	
	N=115	N=25	N=5	EGFR	ALK
**Age(years)**				0.677	0.622
≤60	56	18	3		
>60	59	7	2		
**Gender**				1.000	1.000
Female	69	13	3		
Male	46	12	2		
**Smoking history**				0.636	0.589
Smoking	34	6	2		
Never smoking	81	19	3		
**Tumor size(cm)**					
Median (interquartile range)	3.0 (2.5, 4.0)	3.5 (2.5, 4.5)	2.0 (1.8, 2.7)	**0.017**	**0.011**
≤3	61	11	5	0.063	**0.045**
>3	54	14	0		
**Tumor stage**				1.000	1.000
IIA-IIIA	104	24	5		
IIIB-IV	11	1	0		
**Differentiation**				0.836	0.136
Poor	10	10	0		
Poor-moderate	77	13	4		
Moderate	23	2	1		
Moderate-good	2	0	0		
Undetermined	3	0	0		
**N stage**				0.651	0.642
N1	49	11	3		
N2	66	14	2		

Bold represents statistically significance, $P < 0.05$

[a] P value vs double mutations group
