# Supplementary material for: Gene status and clinicopathologic characteristics of lung adenocarcinomas with mediastinal lymph node metastasis
Source: Oncotarget. 2016 Aug 22;7(39):63758–66. doi: 10.18632/oncotarget.11494 (PMC5325401; doi:10.18632/oncotarget.11494)
Supplement: Supplementary file 2 [file oncotarget-07-63758-s002.doc]

# Table S1 Tumor marker and gene status

| **Tumor marker** | **EGFR(N=120)** | | | | | **ALK(N=30)** | | | **ROS1(N=5)** | | | **RET(N=10)** | | |
| --- | --- | --- | --- | --- | --- | --- | --- | --- | --- | --- | --- | --- | --- | --- |
|  | **Positive**  **(n=120)** | **Negative**  **(n=160)** | | | ***P*** | **Positive**  **(n=30)** | **Negative**  **(n=250)** | ***P*** | **Positive**  **(n=5)** | **Negative**  **(n=275)** | ***P*** | **Positive**  **(n=10)** | **Negative**  **(n=270)** | ***P*** |
| CEA (ng/mL) | 6.8  (2.7,18.4) | | 4.5  (2.2,12.6) | 0.117 | | 4.1  (2.1,9.5) | 5.1  (2.5,17.0) | 0.215 | 2.0  (1.6,6.7) | 5.1  (2.5,15.7) | 0.120 | 3.0  (1.8,23.2) | 5.0  (2.5,15.5) | 0.648 |
| CA199 (U/mL) | 11.8  (6.3,21.5) | | 11.1  (4.7,25.5) | 0.499 | | 15.4  (5.1,31.7) | 11.3  (5.1,22.7) | 0.376 | 4.5  (2.0,9.1) | 11.6  (5.1,25.1) | **0.030** | 18.0  (4.0,51.5) | 11.4  (5.1,23.8) | 0.715 |
| CA125 (U/mL) | 13.2  (9.0,19.4) | | 15.0  (10.1,28.7) | **0.025** | | 15.1  (9.7,34.4) | 13.9  (9.4,21.9) | 0.361 | 12.3  (11.8,15.2) | 14.1  (9.4,22.1) | 0.768 | 16.5  (8.8,51.3) | 14.0  (9.5,22.0) | 0.659 |
| CYFRA21-1 (ng/mL) | 4.7  (3.9,5.0) | | 3.9  (3.3,5.5) | 0.409 | | 4.8  (3.1,7.9) | 4.3  (3.5,4.9) | 0.749 | 3.2  (3.0,3.3) | 4.5  (3.5,5.1) | 0.054 | 3.7  (3.3,4.8) | 4.5  (3.5,5.1) | 0.367 |
| NSE (ng/mL) | 16.2  (12.1,23.1) | | 13.2  (10.3,18.0) | **0.038** | | 11.2  (8.9,19.4) | 14.9  (11.4,20.6) | 0.261 | 7.4  (7.1,7.7) | 14.9  (11.4,20.6) | **0.004** | 12.7  (9.6,21.3) | 14.8  (11.4,20.4) | 0.644 |
| SCCA (ng/mL) | 0.7  (0.4,0.9) | | 0.8  (0.5,1.0) | 0.397 | | 0.9  (0.6,1.1) | 0.7  (0.5,1.0) | 0.449 | 0.4  (0.2,0.6) | 0.7  (0.5,1.0) | 0.128 | 0.8  (0.6,1.6) | 0.7  (0.5,1.0) | 0.434 |

# Abbreviations: CEA, carcinoembryonic antigen; CA199, carbohydrate antigen 199; CA125, carbohydrate antigen 125; CRFRA21-1, cytokeratin fragment 21-1; NSE, neuro specific enolase; SCCA, squamous cell carcinoma antigen

# Number of valid data: CEA, 244; CA199, 241; CA125, 241; CRFRA21-1, 65; NSE, 64; SCCA, 62

# Bold represents statistically significance, *P* <0.05

# Tumor markers are characterized by median values and interquartile ranges
